# Acute Kidney Injury Induced by Systemic Inflammatory Response Syndrome is an Avid and Persistent Sodium-Retaining State

**DOI:** 10.1155/2014/471658

**Published:** 2014-09-21

**Authors:** Daniel Vitorio, Alexandre Toledo Maciel

**Affiliations:** ^1^Intensimed Research Group, Adult Intensive Care Unit, Hospital São Camilo, Pompéia, 05024-000 São Paulo, SP, Brazil; ^2^Intensive Care Unit, Department of Medical Emergencies, Hospital das Clinicas, 05403-001 São Paulo, SP, Brazil

## Abstract

Acute kidney injury (AKI) is a frequent complication of the systemic inflammatory response syndrome (SIRS), which is triggered by many conditions in the intensive care unit, including different types of circulatory shock. One under-recognized characteristic of the SIRS-induced AKI is its avidity for sodium retention, with progressive decreases in urinary sodium concentration (NaU) and its fractional excretion (FENa). This phenomenon occurs in parallel with increases in serum creatinine, being only transitorily mitigated by diuretic use. In the present case, we report a situation of two consecutive shocks: the first shock is hemorrhagic in origin and then the second shock is a septic one in the same patient. The SIRS and AKI triggered by the first shock were not completely solved when the second shock occurred. This could be viewed as a persistent avid sodium-retaining state, which may be appreciated even during renal replacement therapy (in the absence of complete anuria) and that usually solves only after complete AKI and SIRS resolution. We suggest that decreases in NaU and FENa are major characteristics of SIRS-induced AKI, irrespective of the primary cause, and may serve as additional monitoring tools in its development and resolution.

## 1. Introduction

Circulatory shock is a pathological condition resulting from the inadequate tissue perfusion and a mismatch between oxygen supply and demand. Systemic inflammatory response syndrome (SIRS) and subsequent multiple organ dysfunction are a frequent end-point of different types of circulatory shock.

Acute kidney injury (AKI) is one of the most frequent organ dysfunctions in the course of SIRS and shock and carries high morbidity/mortality [1]. The pathophysiological mechanisms involved in the genesis of AKI are frequently not related to hypoperfusion and ischemia [2]. Experimental studies have demonstrated that, during endotoxemia, AKI may develop in parallel with increased renal blood flow [3, 4].

We have recently demonstrated that AKI development is associated with decreases in urinary sodium concentration (NaU) [5] and increases in the fractional excretion of potassium (FEK) [6]; both of them are probably related to activation of the sympathetic and renin-angiotensin-aldosterone systems. During AKI recovery, the opposite phenomena seem to occur.

We have also previously reported the NaU profile in the course of AKI secondary to septic shock [7]. In that case, decreases in NaU were a marker of AKI development, increasing only transitorily after loop diuretic administration and remaining in low levels during the entire AKI course (including renal replacement therapy) until almost complete AKI recovery, when it starts to increase again, returning to baseline levels. The aim of the present case report is to describe the behavior of these same urinary parameters in the course of two consecutive shocks: the first shock is hemorrhagic in origin and then the second one is due to sepsis, both triggering an avid sodium retaining state which seems to characterize SIRS-induced AKI independently of its original source.

## 2. Case Presentation

**Figure 1:**
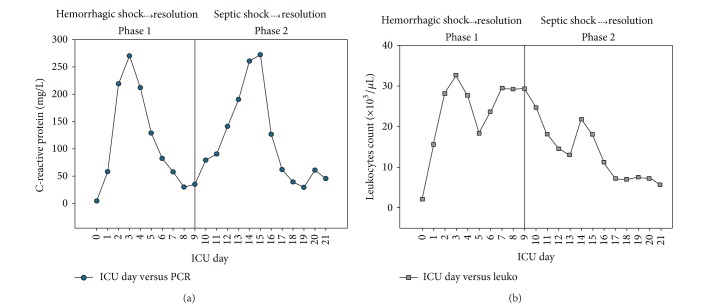
Serum C-reactive protein concentration (a) and leukocytes count (b) in the course of hemorrhagic shock development and resolution (phase 1) and subsequent septic shock development and resolution (phase 2). ICU: intensive care unit.

**Figure 2:**
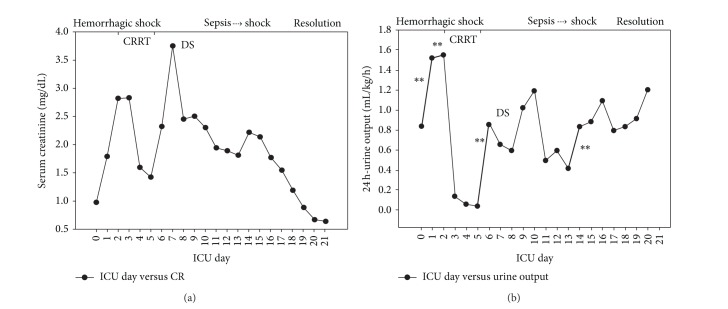
Serum creatinine (a) and urine output (b) in the course of hemorrhagic shock and subsequent septic shock. Bold lines with ∗∗ represent the periods in which furosemide was administered. CRRT: continuous renal replacement therapy DS: conventional dialysis. ICU: intensive care unit.

A 63-year-old female patient was admitted to the ICU in the immediate postoperative period (D0) after being submitted to elective percutaneous lithotripsy, nephrostomy, and left double J stent due to a coraliform stone and recurrent previous urinary tract infections. No complications were reported during the surgical procedure. One hour after admission, the patient developed frank hematuria and bleeding through the nephrostomy tube, circulatory shock, and an abrupt fall of the hemoglobin level from 13 to 6 g/dL. After initial resuscitation with fluids, blood, and vasopressors, she was submitted to embolization of a distal branch of the left renal artery. A subsequent SIRS and multiple-organ dysfunction syndrome developed with abrupt increases in C-reactive protein (CRP—Figure 1(a)), leukopenia followed by leukocytosis (Figure 1(b)), hypothermia (<36°C), metabolic acidosis, hyperlactatemia, need of vasopressors in high doses, mechanical ventilation, and increases in serum creatinine (sCr) (Figure 2(a)).Urine output was maintained with loop diuretic administration (Figure 2(b)). At D2, fever (38.4°C) developed. No infectious agents were retrieved from the cultures at this time. At the end of D2, continuous renal replacement therapy (CRRT) was started due to AKI progression. The clinical condition gradually improved with CRRT, and vasopressors were suspended at D5 in parallel with significant decreases in CRP. At D6, furosemide was administered but at D7 a dialysis session was required due to significant increases in serum urea and sCr. From D8 to D13, renal function gradually improved with spontaneous decreases in nitrogenous waste products and better urine output with no more dialysis sessions required—furosemide was administered only at D13 to prevent a positive fluid balance. At D14, new circulatory shock developed with parallel increases in serum CRP (Figure 1(a)) and procalcitonin (PCT) attributable to a urinary tract infection (*Klebsiella pneumoniae* in uroculture). Vasopressors were again required and new deterioration of renal function occurred (Figure 2), but no RRT was required. After 48 hours of antibiotic therapy the shock reverted with norepinephrine withdrawal and progressive decreases in leukocytes count, CRP (Figures 1(a) and 1(b)) and PCT. In parallel, there was renal recovery, with sCr normalization at D21, the day that the patient was discharged from the ICU.

## 3. Discussion

**Figure 3:**
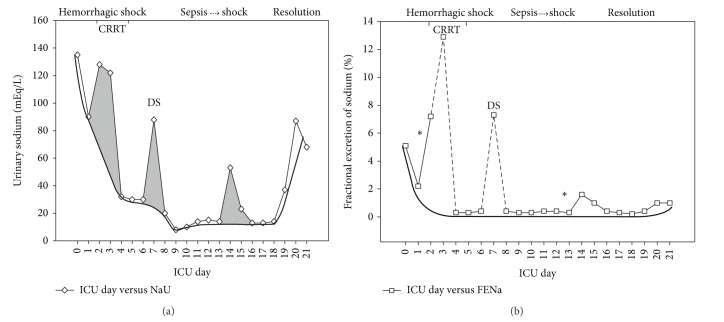
Urinary sodium (NaU) (a) and fractional excretion of sodium (FENa) (b) in the course of hemorrhagic shock and subsequent septic shock. Grey areas represent increases in NaU attributable to furosemide administration. The bold lines represent the hypothetical course of NaU and FENa in the absence of diuretic and CRRT interference. The dashed lines represent the period in which FENa is unreliable due to CRRT. ∗ indicates furosemide administration and its influence in FENa. CRRT: continuous renal replacement therapy DS: conventional dialysis. ICU: intensive care unit.

The above case can be divided in two fundamental parts: the first part (phase 1) that is related to the postoperative hemorrhagic shock and the second part (phase 2) in which the patient has developed a second shock due to a urinary tract infection. In phase 1, AKI developed and rapidly reached an AKIN stage 3 [8] with the need of CRRT. Similar to a previous case report (septic in origin) [7], in the present case, AKI development due to hemorrhagic shock was followed by abrupt decreases in NaU, “artificially” and transitorily increased with furosemide administration (Figure 3(a)). This initial decrease in NaU could be related to hemorrhagic hypovolemia and renal hypoperfusion (a truly prerenal AKI), but it was rapidly corrected so that we believe that decreases in NaU values in the subsequent days are mainly due to shock-induced SIRS.

Since there was no complete anuria during CRRT, we continued to measure NaU and it remained decreasing daily even after RRT interruption. An increase in NaU may be observed between CRRT and conventional dialysis, but this was attributed again to diuretic use. The lowest NaU values were reached only after RRT was completely removed, suggesting a process that was not directly affected by RRT.

If the increases in NaU due to diuretics were ignored (Figure 3(a)), it may be inferred that AKI development was characterized by progressive decreases in NaU which persisted until late in the course of AKI. However, from a different type of shock, this behavior is very similar to that of our previous case report [7] and emphasizes that low NaU is a common characteristic of SIRS-induced AKI development even in those with persistent AKI and need of CRRT. This argues against the old concept of “persistent” as a synonym for “structural” or “acute tubular necrosis.” NaU profile behaved all this period as a progressive “prerenal” AKI and this is probably a reflection of decreases in glomerular filtration rate (GFR) together with an avid capacity of the tubules to retain sodium, which may occur even in the absence of renal hypoperfusion [4].

In the previous case [7], NaU started to recover only when almost normal values of sCr and CRP were reached. In the present case, there was also a peak of CRP and leukocytes around D3 (Figure 1) suggesting an inflammatory component triggered by the hemorrhagic shock. CRP was then decreasing, but at D9 a new increase was triggered by sepsis (phase 2). We believe that this phenomenon has prevented AKI recovery to continue so that sCr had a small but new increase at D14 and NaU remained in low levels, “artificially” increasing after furosemide administration (Figure 3(a)) but decreasing again soon after and truly recovering only when the second CRP peak and leukocytosis (the second inflammatory booster) were solved with sepsis treatment. In fact, low NaU levels seem to be tightly related to the presence of SIRS in critically ill patients, infectious [7] or noninfectious [9] in origin. Significant increases in NaU in the last ICU days in the absence of diuretics were probably a combination of increased sodium filtration and decreased tubular sodium reabsorption (SIRS resolution).

The behavior of FENa reaffirms the avid sodium retention that was triggered by shock/SIRS (Figure 3(b)). FENa value decreased abruptly (similar to NaU) and was also only temporarily increased by diuretic use. In addition, FENa interpretation during RRT is unreliable. Again, ignoring the periods of diuretic use and RRT, there was a clear descending curve of FENa with “real” (although discrete) increases only in the late phase of AKI recovery, compatible with less avidity for sodium reabsorption secondary to less activity of sympathetic nervous and renin-angiotensin-aldosterone systems. These two systems seem to have a pivotal role in the AKI genesis [10], interfering in both GFR and electrolyte reabsorption/secretion in the tubules.

**Figure 4:**
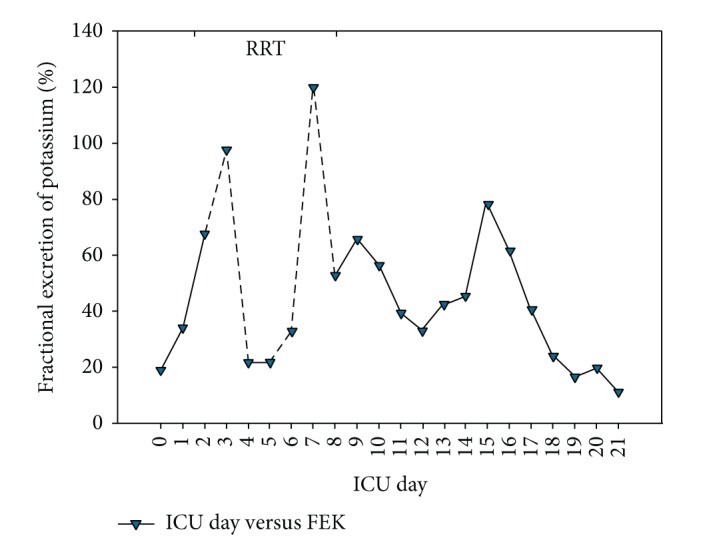
Fractional excretion of potassium (FEK) in the course of hemorrhagic shock and subsequent septic shock. Dashed lines represent the period in which FEK is unreliable due to renal replacement therapy (RRT). ICU: intensive care unit.

Since FENa usually has a low range of variation (except after diuretic), we have proposed the evaluation of FEK [6] in the course of AKI since sodium and potassium usually have opposite behaviors in AKI. Discrete decreases in FENa may be followed by more “visible” increases in FEK. However, similar to FENa, FEK also increases after loop diuretic administration [11]. Hence, interpretation of its value must be careful. In the present case, FEK value was already increased at ICU admission (around 20%, normal value 10%), suggesting some degree of stress to the kidneys which could be due to the surgical procedure itself (Figure 4). Increases in FEK occurred from D0 to D1 following increases in sCr. This increase was exacerbated by furosemide administration. Fractional excretion values are not real during RRT. Abrupt decreases in FEK in this period were, in our view, only the result of a mathematical coupling with sCr, which decreased abruptly during CRRT. It is noteworthy that the FEK curve was very similar to that of sCr, decreasing and increasing together (Figures 2(a) and 4). This phenomenon did not seem to be merely “mathematical” in the absence of RRT since FENa curve did not follow sCr in the same way. Near-normal values of FEK were only reached at the last ICU days (similar to NaU) and usually after normalization of sCr. We believe, based on this case and our previous studies [5, 6], that both FEK and NaU may begin to change before sCr in AKI development and reach normal values only after complete sCr normalization. Therefore, these parameters have great potential to be valuable tools in AKI monitoring.

## 4. Conclusion

Circulatory shocks, independently of their origin, trigger similar alterations in the urinary biochemical profile, particularly in NaU, FENa, and FEK. These alterations may precede increases in sCr and remain even after its normalization. Persistent AKI may have a persistent “prerenal” pattern in inflammatory states suggesting that decreases in GFR are followed by preserved global tubular capacity to avidly reabsorb sodium even in advanced stages of AKI. Significant increases in NaU in the absence of diuretics may be a sign not only of AKI resolution but also of SIRS resolution. However, further studies are required to better define the role of these urinary biochemical parameters in the context of SIRS generated by different types of shock.
